# Self-Assembled Few-Layered MoS_2_ on SnO_2_ Anode for Enhancing Lithium-Ion Storage

**DOI:** 10.3390/nano10122558

**Published:** 2020-12-20

**Authors:** Thang Phan Nguyen, Il Tae Kim

**Affiliations:** Department of Chemical and Biological Engineering, Gachon University, Seongnam-si, Gyeonggi-do 13120, Korea; phanthang87@gmail.com

**Keywords:** SnO_2_, self-assembly, MoS_2_, nanosheets, lithium-ion battery

## Abstract

SnO_2_ nanoparticles (NPs) have been used as reversible high-capacity anode materials in lithium-ion batteries, with reversible capacities reaching 740 mAh·g^−1^. However, large SnO_2_ NPs do not perform well in charge–discharge cycling. In this work, we report the incorporation of MoS_2_ nanosheet (NS) layers with SnO_2_ NPs. SnO_2_ NPs of ~5 nm in diameter synthesized by a facile hydrothermal precipitation method. Meanwhile, MoS_2_ NSs of a few hundreds of nanometers to a few micrometers in lateral size were produced by top-down chemical exfoliation. The self-assembly of the MoS_2_ NS layer on the gas–liquid interface was first demonstrated to achieve up to 80% coverage of the SnO_2_ NP anode surface. The electrochemical properties of the pure SnO_2_ NPs and MoS_2_-covered SnO_2_ NP anodes were investigated. The results showed that the SnO_2_ electrode with a single-layer MoS_2_ NS film exhibited better electrochemical performance than the pure SnO_2_ anode in lithium storage applications.

## 1. Introduction

The use of lithium-ion batteries (LIBs) helps to solve issues of energy and powering devices in modern life and industries. The prevalence of electronic devices in life and the rapid development of industry have led to a demand for higher-quality energy storage systems with improvements such as higher capacity, greater stability, and improved safety. Currently, commercialized LIBs use lithium–nickel–manganese–cobalt oxide cathodes, which are generally stable but difficult to improve, and graphite anodes, which have a low capacity of ~372 mAh·g^−1^ [1,2]. The issues requiring attention in the development of LIBs are low anode capacity, degradation, and mechanical instability in LIB structures due to anode material expansion by the insertion and extraction of lithium ions. To improve LIB characteristics, several anode materials have been considered, such as metal alloys, metal oxides, and transition metal chalcogenides (TMCs) [3,4,5]. Recently, the use of metal oxide materials such as TiO_2_, SnO_2_, Fe_2_O_3_, and V_2_O_5_ for lithium-ion storage anodes has attracted much attention owing to their chemical stability and high reversible capacity [6,7,8,9,10,11,12,13,14,15]. Among them, SnO_2_ nanoparticles (NPs) have shown promising lithium-ion storage capability with very high capacity reaching 740 mAh·g^−1^ [16,17]. It has been reported that the required SnO_2_ material size is approximately 3 nm to achieve a high reversible capacity [16]. However, the lithiation process of SnO_2_ leads to large volume expansion, which results in poor reversible cycling performance [18]. Many methods have been employed to enhance the reaction stability of SnO_2_ materials by using carbon-based support materials such as amorphous carbon or graphene, as well as MoS_2_. For example, Lou et al. used carbon-supported SnO_2_ nanocolloids prepared by a facile hydrothermal method and carbonization [19]. Wang et al. developed a SnO_2_–graphene composite material for high-capacity reversible LIBs, which had a capacity of ~440 mAh·g^−1^ after 100 cycles [20]. Chen et al. reported a composite of SnO_2_ and MoS_2_ nanosheets (NS) as a LIB anode to diminish the volume expansion during lithiation in SnO_2_ NPs [21]. However, the appropriate working conditions and optimization of the SnO_2_ morphologies still require investigation and remain challenging in realizing commercial batteries.

Along with the development of metal oxide materials, 2D materials such as graphene and TMCs have drawn great attention owing to their superior properties and flexibility [22,23,24,25,26]. Single- and few-layered TMCs, including MoS_2_ and WS_2_, have revealed superior electronic and mechanical properties. They have also been utilized in many optical and electrical devices such as solar cells, light-emitting diodes, and transistors, as well as applications in catalysts for hydrogen generation and energy-storage applications in LIBs or sodium-ion batteries [27,28,29,30]. MoS_2_ in 1T phase has been used to overcome charge–discharge decay and to effectively store lithium or sodium ions with the aid of carbon derivatives such as graphite, carbon nanotubes (CNTs), and graphene. For instance, a combination of CNTs and 1T-structured MoS_2_ reported by Nguyen et al. [14] was introduced to develop 3D MoS_2_@graphite-CNT for a long-term stable anode material with a very high lithium-ion storage capacity. The 3D-structured MoS_2_@gaphite-CNT was prepared by a scalable ball-milling method. This 3D structure allowed a high anodic charge–discharge rate and showed a high capacity of ~1200 mAh·g^−1^ after 450 cycles. Moreover, Lane et al. reported the computed electronic structure of lithium- and sodium-intercalated MoS_2_. They concluded that the 1T-MoS_2_ could be a high-capacity and high-conductivity anode material [31]. Li et al. also discovered that the growth of 3D bulky MoS_2_ in the 1T phase supported prolonged anodic cycling, where the structure helped to release strain during cycling and led to improved capacity at high current rates [32]. Therefore, the use of 1T-MoS_2_, which has high conductivity and flexibility, can help traditional anode materials exhibit superior performance in lithium storage applications.

In this work, we first demonstrated the use of a 1T-MoS_2_ self-assembling layer as protection on the SnO_2_ surface. The MoS_2_ NS layer was formed by using the gas–liquid interface method, which is well-known for the thin-film preparation of monolayer colloidal crystals such as polystyrene NPs [33,34,35]. SnO_2_ NPs were fabricated by a facile hydrothermal method. The presence of the 1T-MoS_2_ layer greatly enhanced the cycling performance of SnO_2_ anodes in LIBs. Moreover, the effect of different numbers of MoS_2_ layers was also investigated.

## 2. Experimental

### 2.1. Chemical Materials

Molybdenum(VI) sulfide (MoS_2_, powder), tin(II) chloride dihydrate (SnCl_2_·2H_2_O, powder), polyvinylidene fluoride (PVDF, M_W_ 534,000), Sodium dodecylbenzensulfonate (technical grade) and a 2.5-M solution of *n*-butyllithium in hexane were purchased from Sigma-Aldrich Inc. (St. Louis, MO, USA). Super-P amorphous carbon black (C, approximately 40 nm, 99.99%) and absolute ethanol (C_2_H_5_OH) were purchased from Alpha Aesar Inc. (Ward Hill, MA, USA). All chemicals were used as delivered without any purification.

### 2.2. Exfoliation of MoS_2_ Nanosheets (NSs)

MoS_2_ NSs were synthesized using the liquid chemical exfoliation method [36,37,38]. In brief, 1 g of MoS_2_ powder was placed in a 10-mL vessel. Then, 3 mL of butyllithium in hexane was added to reach the powder level. The solution was kept for 2 days to allow lithium intercalation into the MoS_2_ to form Li*_x_*MoS_2_ (*x* > 1) [36,39]. For the exfoliation of MoS_2_, Li*_x_*MoS_2_ was washed with hexane to remove excess butyllithium by centrifugation at 5000 rpm for 5 min. The Li*_x_*MoS_2_ obtained was exfoliated in 100 mL deionized (DI) water in a sonication bath for 1 h. The floating large-sized MoS_2_ in the solution was removed. Then, 1T-MoS_2_ was centrifuged with DI water four times and kept in DI water for further use or dried at 60 °C in a vacuum oven for characterization.

### 2.3. SnO_2_ Nanoparticle (NP) Synthesis

SnO_2_ NPs were prepared by a facile hydrothermal method. In a typical synthesis, 0.9 g of SnCl_2_·2H_2_O was dissolved in 100 mL of DI water under stirring for 30 min. Then, 10 mL of 10% ammonia solution (NH_4_OH) was dropped slowly to obtain a gel form. The solution was then transferred to a Teflon autoclave line and heated at 200 °C for 2 h. The white precipitate, SnO_2_, was collected via centrifugation and washing for 3 times, then dried and calcinated in air at 600 °C for 1 h.

### 2.4. Self-Assembled MoS_2_ NS Layer

**Figure 1:**
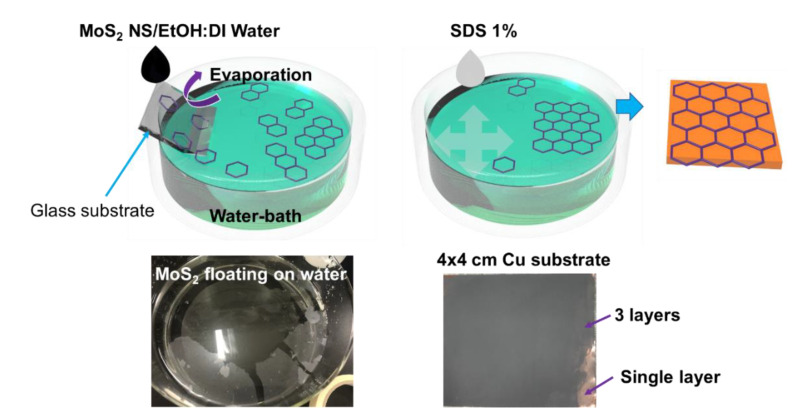
Illustration and photographs of self-assembled MoS_2_ nanosheet (NS) on Cu substrate.

The self-assembled MoS_2_ was prepared based on the wettability of the MoS_2_ NSs. MoS_2_ NSs can be dispersed in DI water; however, an NS floats on the surface of DI water if only one NS surface is wetted. This behavior is similar to that of graphene or polystyrene, both of which can assemble as 2D layers floating on a solution. Here, MoS_2_ NSs were dispersed in a 1:1 volumetric DI water–ethanol mixture at a concentration of 2 mg·mL^−1^. A water bath and half-dipped glass substrate are shown in Figure 1. The prepared solution was then dropped slowly to allow evaporation of the DI water–ethanol solution as the drop met the water surface. The MoS_2_ NSs remained randomly floating at the water–air interface. To consolidate the single-layer MoS_2_ NSs, a ~10-μL drop of 1% sodium dodecylbenzensulfonate solution was spread on the surface to increase the surface tension. This formed a semi-transparent layer of MoS_2_ on the side of the water bath. The dried anode material was dipped in DI water before it was dipped in the assembled MoS_2_ NS layer bath. The MoS_2_ NS layers were easily deposited on the electrode surface. Finally, the anode was obtained by drying in a vacuum oven at 70 °C.

### 2.5. Characterization

X-ray diffraction (XRD) (D/MAX-2200 Rigaku, Tokyo, Japan) was used to investigate the structures of the powder samples. The XRD patterns of the samples were performed over the 2θ range of 10−70°. The structures, morphologies, and sizes of the materials were analyzed by scanning electron microscopy (SEM) (Hitachi S4700, Tokyo, Japan) and transmission electron microscopy (TEM) integrated with energy-dispersive X-ray spectroscopy (EDS) (TECNAI G2F30, FEI Corp., OR, USA).

### 2.6. Electrochemical Measurements

The SnO_2_ NP materials were employed to assemble a half-cell LIB using coin-type cells (CR 2032, Rotech Inc., Gwangju, Korea). The assembly process follows a typical LIB construction method. The working electrode was prepared by pasting a slurry of 70% active material (SnO_2_ NP powder), 15% PVDF, and 15% Super P in *N*-methyl-2-pyrrolidinone on a Cu foil by doctor blading. The electrode was then dried in a vacuum oven at 70 °C for at least 12 h before use. The MoS_2_ NS layer was lifted from the water surface, as mentioned before, and dried before cell assembly. Electrodes with one, two, and three MoS_2_ NS layers were denoted as M1SnO_2_, M2SnO_2_, and M3SnO_2_, respectively. The anodes were punched into circular discs of 12 mm in diameter. The areal loading of active materials was 0.84–1.05 mg cm^−2^. The battery half-cell structures were assembled under a neutral gas of Ar in a glovebox with positive pressure to ambient air conditions. The reference electrode, separator, and electrolyte were lithium foil, polyethylene, and 1-M LiPF_6_ in ethylene carbonate–diethylene carbonate (1:1 by volume), respectively. The galvanostatic electrochemical charge–discharge performances of the different cells were measured using a battery cycle tester (WBCS3000, WonAtech, Seocho-gu, Seoul) over the voltage range of 0.01–3.0 V versus Li/Li^+^. Cyclic voltammetry (CV) tests and electrochemical impedance spectroscopy (EIS) were performed using a ZIVE MP1 (WonAtech, Seocho-gu, Seoul) over the voltage range of 0.01–3.0 V at a scanning rate of 0.1 mV·s^−1^ and over the frequency range of 100 kHz–0.1 Hz.

## 3. Results and Discussion

**Figure 2:**
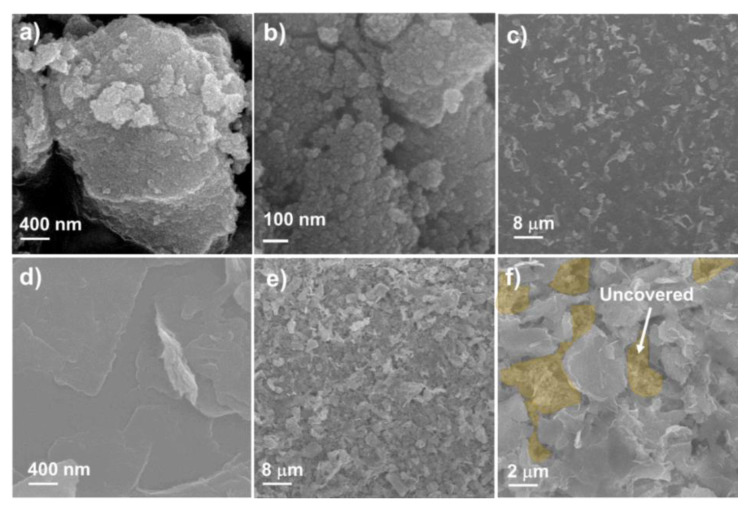
Scanning electron microscope (SEM) images of (**a**,**b**) SnO_2_ nanoparticle (NP) powder, (**c**,**d**) single-layer MoS_2_ NS thin film on Cu electrode, and (**e**,**f**) M1SnO_2_ anode.

Figure 2 shows the SEM images of the SnO_2_ NP, MoS_2_ NS, and SnO_2_ anode covered with the MoS_2_ NS layer. To prepare the SnO_2_ NPs, an easy hydrothermal method was selected to obtain a uniform and highly crystalline material [40,41,42]. Sn(OH)*_x_* precipitation using NH_4_OH solution and hydrothermal processing were applied to develop the crystalline structures of the SnO_2_ NPs. It was observed that the size of the NPs was <100 nm. However, the detailed size distribution of the NPs cannot be determined by SEM measurement, as illustrated in Figure 2a,b. The diameters of the SnO_2_ NPs are discussed later via TEM analyses. Meanwhile, the MoS_2_ NSs were well prepared with lateral sizes ranging from a few hundred nanometers to a few micrometers, as shown in Figure 2c,d. The size of the NSs is larger than that of MoS_2_ fabricated by ultrasonication or ball milling [43,44]. The low-magnification SEM image shows that the MoS_2_ NSs uniformly cover the Cu electrode. Figure 2e,f shows a single-layer MoS_2_ NS thin film on the surface of the SnO_2_ electrode. It was calculated that the MoS_2_ NSs covered approximately 80% of the SnO_2_ electrode surface. The semi-transparent yellow indicates the uncovered electrode surface (Figure 2f), while the dark colors indicate the thin MoS_2_ layer covering the surface. From analysis of the SEM images, a novel SnO_2_ electrode uniformly covered with MoS_2_ NSs layers was prepared, from which better electrochemical performance is expected, compared to that of a pure SnO_2_ electrode.

**Figure 3:**
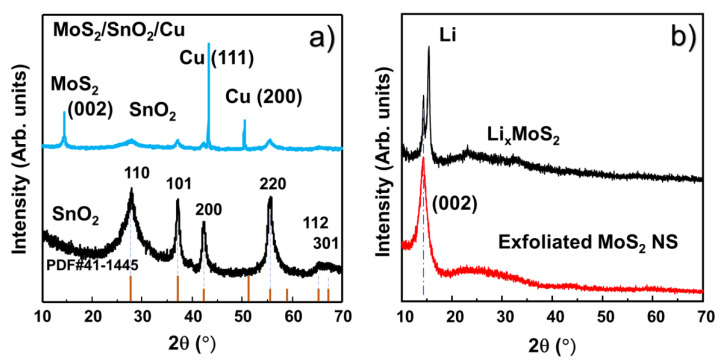
X-ray diffraction (XRD) patterns of (**a**) SnO_2_ NPs and M3SnO_2_ anode and (**b**) lithium-ion intercalated and exfoliated MoS_2_ nanosheet.

To confirm the crystalline nature of the SnO_2_ NPs, powder XRD measurements were performed in the range of 10–70°, as shown in Figure 3a. In comparison with PDF #41-1445, the XRD pattern of the SnO_2_ NPs was in good agreement with the P4_2_/mnm space group [16]. No impurity peaks appear in the pattern. This confirmed the high crystallinity of the SnO_2_ NPs. Furthermore, the average sizes of the crystals (D) can be calculated using the Scherrer equation:

D = 0.9*λ*/*β*cos*θ* (1)

where *λ* is the X-ray wavelength of the XRD measurement, *θ* is the Bragg angle, and *β* is the full width at half maximum of the peak. Based on this information, the calculated crystal size of the SnO_2_ NPs was ~2–5 nm. The structure of the assembled MoS_2_ layer on SnO_2_ anode was also analyzed by XRD as shown in Figure 3a. The peaks of MoS_2_ NS, SnO_2_ and Cu were clearly revealed, indicating successful coverage of MoS_2_ on the SnO_2_ anode. Figure 3b shows the XRD patterns of Li*_x_*MoS_2_ and the MoS_2_ NSs. The intercalation of lithium in the interface of each layer MoS_2_ introduced a peak at ~15.4°. After exfoliation in DI water, only the peak of (002) MoS_2_ remained, indicating the 2D structure of the MoS_2_ NSs. The size of MoS_2_ based on the (002) plane was calculated to be ~1 nm, illustrating the formation of thin and single- or few-layered MoS_2_ NSs [37,38]. The nanoscale sizes of the SnO_2_ NPs are promising for achieving the critical size of SnO_2_ that yields the best electrochemical performance. Moreover, high-quality single- and few-layer MoS_2_ with large lateral sizes effectively cover the electrode surface.

**Figure 4:**
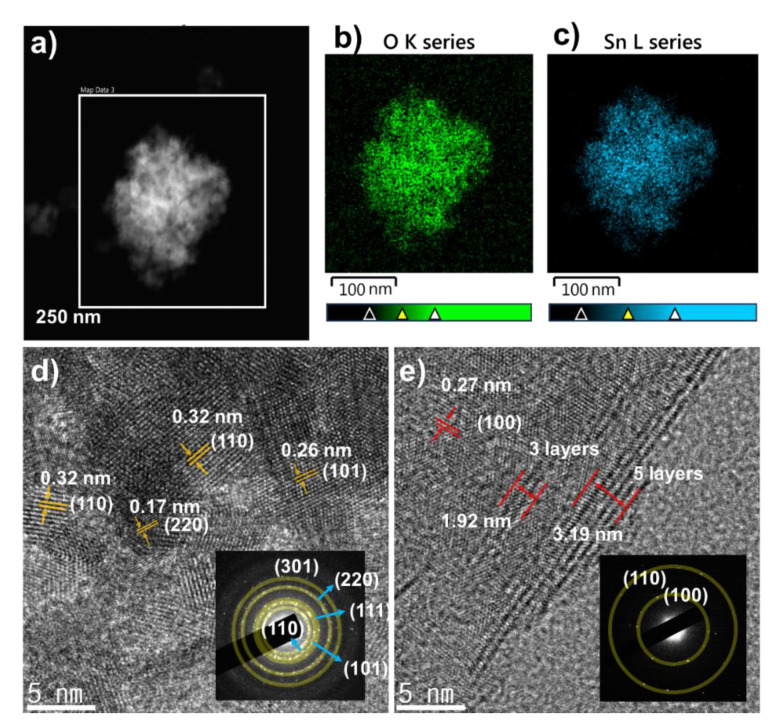
(**a**) Transmission electron microscope (TEM) image of SnO_2_ NP; energy-dispersive X-ray spectroscopy (EDS) mapping of the elements (**b**) O and (**c**) Sn; (**d**) high-resolution TEM (HRTEM) image of SnO_2_ NP with inset selected-area electron diffraction (SAED) pattern; (**e**) exfoliated MoS_2_ NS with inset SAED pattern.

To further confirm the sizes and structures of the SnO_2_ NPs, the SnO_2_ powder was observed by TEM. Figure 4a–c shows the SnO_2_ EDS mapping images, where clear contrasts indicating Sn and O atoms were detected, indicating the high purity of the synthesized materials. In addition, the high-resolution TEM (HRTEM) image (Figure 4d) clearly shows the lattices of SnO_2_ NPs with (101), (110), and (220) planes with sizes of ~5 nm. This result agrees well with the calculation of the SnO_2_ crystal size from the XRD patterns. Furthermore, the selected-area electron diffraction (SAED) pattern in Figure 4d also confirms the lattice planes from the crystal structures, as marked with semi-transparent yellow circles. No other elements were found as contaminants. Therefore, pure SnO_2_ NPs were well prepared with a diameter of ~5 nm, which would contribute to a high performance in LIBs. Meanwhile, the morphology of MoS_2_ NS was also confirmed by HRTEM (Figure 4e), where a lattice spacing of 0.27 nm can be assigned to (100) plane. The spacing between lattice fringes in HRTEM image were to be 1.92 and 3.19 nm for 3 and 5 layers of MoS_2_, respectively. Furthermore, the SAED as an inset of Figure 4e also confirmed the high crystallinity of the MoS_2_ layer. Therefore, it can be considered that the MoS_2_ NS was successfully exfoliated to single- and few-layer MoS_2_.

To understand the effect of the MoS_2_ NS layer on the electrochemical properties of the SnO_2_ anode, CV tests were performed between 0.01 and 3.00 V (vs. Li/Li^+^) at a scanning rate of 0.1 mV·s^−1^. The electrochemical process in the anode can be expressed by the following reaction equations:

(2) $${\mathrm{Li}}^{+}\;+{\;e}^{-}\;+\;\mathrm{electrolyte}\;\to \mathrm{SEI}\;\mathrm{layer}$$

(3) $${\mathrm{SnO}}_{2}+4{\mathrm{Li}}^{+}+4e^{-}\to \mathrm{Sn}+2{\mathrm{Li}}_{2}O$$

(4) $$\mathrm{Sn}+x{\mathrm{Li}}^{+}+xe^{-}↔{\mathrm{Li}}_{x}\mathrm{Sn}\;(0\leq x\leq 4.4)$$

**Figure 5:**
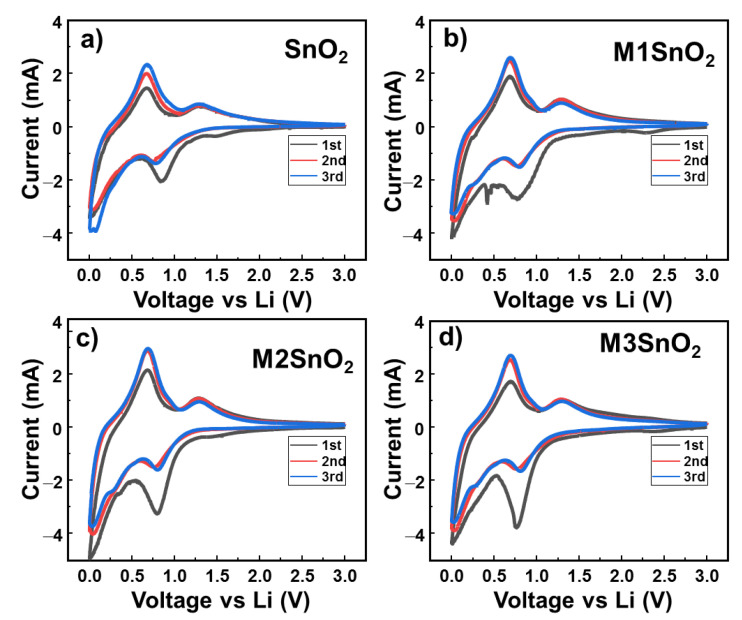
Cyclic voltammetry (CV) profiles of (**a**) SnO_2_ NS and (**b**–**d**) M1/M2/M3SnO_2_ anodes over three cycles.

Equation (2) relates to the formation of a solid electrolyte (SEI) layer formed from the first lithium-ion insertion. Meanwhile, Equation (3) is the conversion reaction of SnO_2_ to Sn. Both reactions (2) and (3) contribute to the irreversible capacity of the anode. Reaction (4) represents the reversible reaction of Sn with lithium ions. Figure 5a shows the electrochemical performance of the pure SnO_2_ anode in the initial three cycles. The peak at ~0.84 V in the first cathodic cycle is attributed to the reduction of SnO_2_ to Sn metal and the formation of the SEI layer, as illustrated in Equations (2) and (3). In the 2nd and 3rd cycles, these peaks showed reduced intensity and were shifted to the lower potential of ~0.75 V. In the oxidation process, two peaks appear at approximately 0.67 and 1.30 V. Interestingly, the oxidation peak at 0.67 V increases in intensity as the cycle number was increased. This phenomenon could be explained by the activation of the reversible reaction that occurred in the electrode materials [17]. Meanwhile, the oxidation peak at 1.30 V was ascribed to the oxidation of metallic Sn to SnO_2_, which is the reversible case of reaction (2) [17,20,45]. When the MoS_2_ NS layer was added to the SnO_2_ anode surface, the CV profiles changed depending on the number of layers, as shown in Figure 5b–d, respectively. First, it is easily observed that the oxidation peaks remain stable, with two peaks at 0.67 and 1.30 V related to the reversible reduction and oxidation reaction of Sn metal. With the M1SnO_2_ electrode, additional peaks appeared at ~0.42 V and 0.45 V, arising from the conversion reaction as: Li*_x_*MoS_2_ + (4 − *x*) Li^+^ + (4 − *x*) e^−^ → Mo + 2 Li_2_S and from SEI layer formation on the MoS_2_ materials [46,47,48,49]. The M2/M3SnO_2_ electrodes also have peaks at ~0.42 V; however, the intensities are weaker than that of the M1SnO_2_ electrode. This can be explained by the energy barrier of the single-layer MoS_2_ for lithium-ion intercalation, which is in the range of ~0.42 to ~0.16 eV. Meanwhile, bulk or multilayer MoS_2_ has an intercalation energy barrier between 0.73 and 0.59 eV. Therefore, the peaks in the M2/M3SnO_2_ electrodes were divided by a small peak at 0.42 V and a joined peak in the range of 0.75–0.84 V of SnO_2_ [50]. In addition, the peak intensity at ~0.8 V related to the reduction of SnO_2_ increased compared to those of the pure SnO_2_ and M1SnO_2_ electrodes because of the joined peak of the MoS_2_ multilayer. Therefore, it is thought that the addition of MoS_2_ NS (two and three layers) might lead to the formation of a large SEI layer. Further, the direct contact of each NS layer can increase the possibility of restacking of the MoS_2_ NSs, leading to thicker and bulky MoS_2_ NS structures.

**Figure 6:**
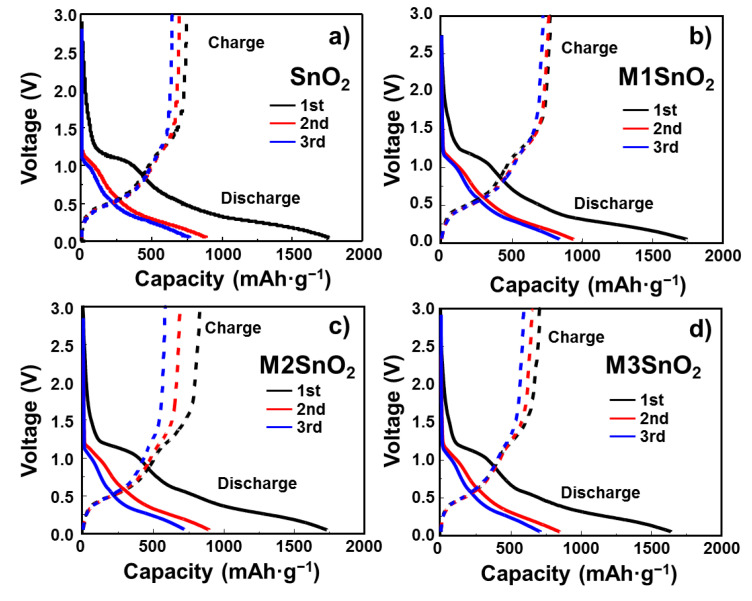
Galvanostatic charge–discharge profiles of (**a**) SnO_2_ NS and (**b**–**d**) M1/M2/M3SnO_2_ anodes for the initial three cycles.

The initial voltage profiles of the SnO_2_ and M1/M2/M3SnO_2_ electrodes at 100 mA·g^−1^ from 0.01 to 3.0 V are shown in Figure 6. The initial discharge capacity of SnO_2_ showed a very high value of ~1760 mAh·g^−1^. This was dramatically reduced to ~880 and ~760 mAh·g^−1^ for the 2nd and 3rd discharges, respectively, indicating a large irreversible reaction due to the formation of an SEI layer and an initial coulombic efficiency (ICE) of 42.3%. However, in the case of the M1SnO_2_ electrode (Figure 6b)**,** the 1st, 2nd, and 3rd cycles exhibited slow decreases in the discharge/charge capacities from 1740/760 mAh·g^−1^ to 932/755 mAh·g^−1^ and 835/710 mAh·g^−1^, respectively. Figure 6c shows the voltage profile of the M2SnO_2_ electrode. It shows the 1st discharge/charge capacity of 1730/823 mAh·g^−1^, corresponding to an ICE of 47.6%. Therefore, it is thought that the better coverage of the MoS_2_ NS double layer improved the charge capacity from 760 to 823 mAh·g^−1^ compared to that of the M1SnO_2_ electrode in the first cycle. However, from the next cycle, the capacity was reduced rapidly to 580 mAh·g^−1^ in the 3rd cycle. Similarly, the additional coating layer on SnO_2_ (M3SnO_2_) caused a significant reduction in the discharge/charge capacity in the 1st cycle to 1640/701 mAh·g^−1^, which was decreased to ~700/580 mAh·g^−1^ in the 3rd cycle. In summary, the addition of MoS_2_ layers to the SnO_2_ electrode improved the electrochemical performance. However, thicker MoS_2_ NSs in excess of two layers decreased the lithium storage capability. This could be explained by the restacking of the MoS_2_ NS layers, leading to the formation of bulky or thicker layers. Moreover, TMC materials such as MoS_2_ and WS_2_ have been reported to improve the electronic properties when present as very thin single- or few-layer structures [31]. This information is in good agreement with the single-layer MoS_2_ NS-coated SnO_2_ meeting the requirements of enhancing the storage ability, whereas multilayer MoS_2_ NS-coated SnO_2_ showed faster degradation in battery performance.

**Figure 7:**
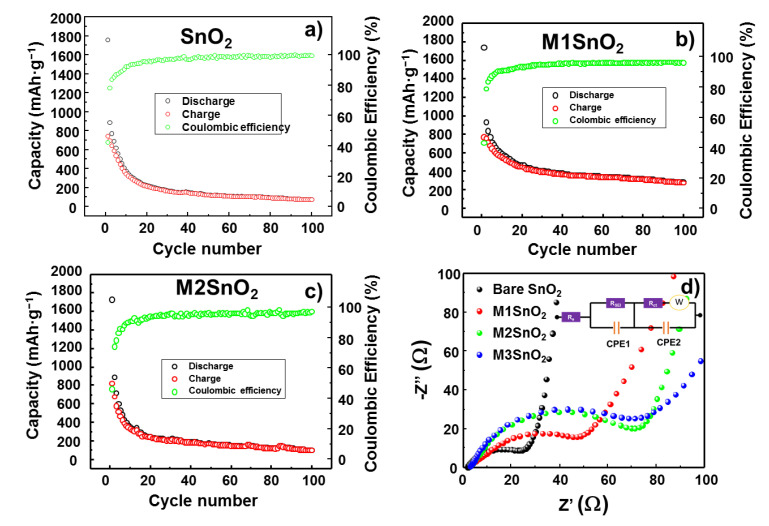
Cyclic performances and Nyquist plots of (**a**) SnO_2_ NPs and (**b**–**d**) M1/M2/M3SnO_2_ anodes.

To evaluate the long-term cyclability, the LIB half-cell with and without the MoS_2_ NS layer on the SnO_2_ surface were subjected to 100 charge–discharge cycles at a current rate of 100 mA·g^−1^, as shown in Figure 7a−c. The mass in all measurements was calculated as the real mass of active materials, including SnO_2_ and MoS_2_, in the electrode. The pure SnO_2_ anode showed a substantial decrease in the discharge/charge capacity from 1750/745 mAh·g^−1^ to 373/345 mAh·g^−1^ at the 10th cycle and to 74.4/73.9 mAh·g^−1^ at the 100th cycle, as illustrated in Figure 7a. With the addition of the MoS_2_ NS layer, the cyclic stability of lithium storage was much higher than that of the bare electrode. With a similar initial discharge/charge capacity of 1740/760 mAh·g^−1^, at the 10th cycle, they exhibited those of 593/551 mAh·g^−1^ and retained those of 281/277 mAh·g^−1^ at the 100th cycle. It is well observed that the coulombic efficiency retained a high value of ~99%, as shown in Figure 7b. The electrode with >2 layers of MoS_2_ NSs showed the worst cyclic performance, retaining a discharge/charge capacity of only 103/102 mAh·g^−1^ at the 100th cycle (Figure 7c). Furthermore, the charge-transfer resistance of the cells was measured from the EIS analysis, as depicted in Figure 7d. The modified Randles equivalent circuit was determined to contain a series resistance (R_s_), a charge-transfer resistance (R_ct_), an SEI resistance (R_SEI_), and a Warburg impedance element (W) (Figure 7d inset) [4]. The extracted values of these resistances are shown in Table 1. It is easy to observe that the bare SnO_2_ has the lowest charge-transfer resistance of ~23.86 Ω, while an increasing number of MoS_2_ NS layers corresponded to increased charge-transfer resistance in the electrodes. In contrast, the R_SEI_ was dramatically reduced from 500.2 Ω in the SnO_2_ NP electrode to 182.7 Ω in the M1SnO_2_ electrode, and then slightly increased to 284.8 Ω and 305.1 Ω for the M2 and M3SnO_2_ electrodes, respectively. The MoS_2_ layer provides a large uniform surface compared to that of bare SnO_2_, thus reducing R_SEI_. Moreover, as discussed above, the electrode with single-layer MoS_2_ has a low lithium intercalation energy barrier of ~0.42 eV, while the electrodes with thicker layers have the higher energy barrier of ~0.73 eV. This could lead to an increase in the R_SEI_. Based on the aforementioned results, the single layer of MoS_2_ NS is important in reducing the SEI resistance, thus helping the long-term cycling stability of the SnO_2_ anode for lithium storage. This study suggests that coverage of SnO_2_ NPs with a thin and complete large-scale MoS_2_ layer can significantly enhance the electrode performance in LIBs.

**Figure 8:**
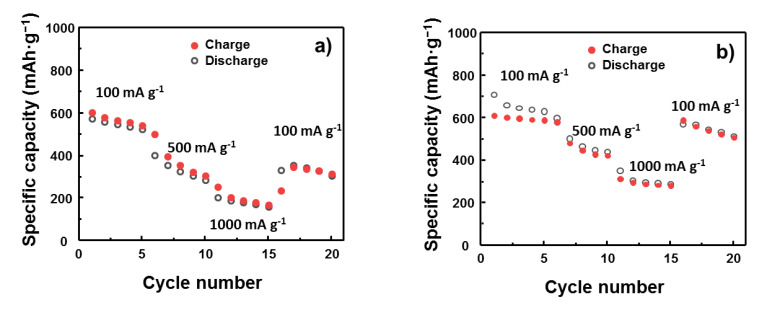
Rate cycling performance of (**a**) bare SnO_2_ electrode and (**b**) M1SnO_2_ electrode at different current rates.

The rate cycling performance of the bare SnO_2_ and M1SnO_2_ electrodes was also investigated, as shown in Figure 8. Performance was recorded for 20 cycles each at charge rates of 100, 500, and 1000 mA·g^−^^1^ before the charge rate returning to 100 mA·g^−^^1^. The SnO_2_ electrode shows greater degradation in specific capacity retention when the scan rate is increased. The retentions after 5 cycles at 500 and 1000 mA·g^−1^ were 47% and 26%, respectively. In contrast, the M1SnO_2_ electrode shows a smaller decrease in specific capacity retention compared to that of pure SnO2, where the retentions at 500 and 1000 mA·g^−1^ were 70% and 47%, respectively, thus demonstrating much better rate performance than the SnO_2_ electrode did. The restoration of the M1SnO_2_ electrode when the current rate was decreased from 1000 to 100 mA·g^−1^ was also remarkable, corresponding to 95%, far exceeding that (55%) of the SnO_2_ electrode. These results suggest that the introduction of a single MoS_2_ layer on SnO_2_ electrodes could further enhance the electrochemical performance.

**Figure 9:**
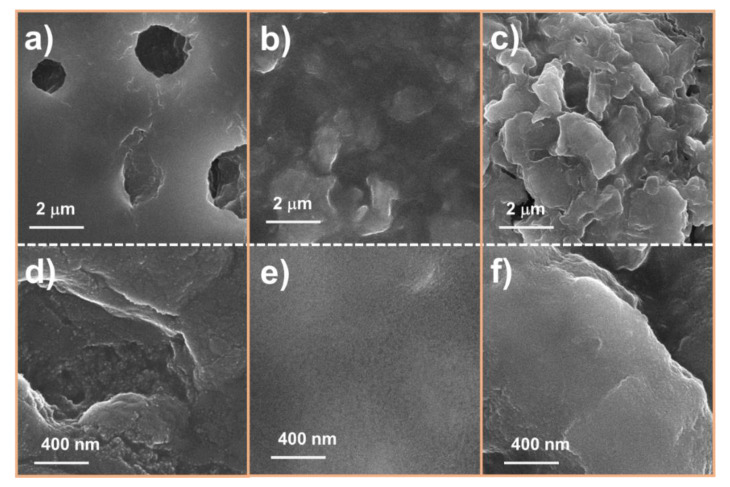
SEM images of (**a**,**d**) SnO_2_ electrode, (**b**,**e**) M1SnO_2_ electrode and (**c**,**f**) M3SnO_2_ electrode after 10 cycles at different magnifications.

To further confirm the effect of the MoS_2_ layer, the surface of the electrode was characterized by ex situ SEM, as shown in Figure 9. The assembled half-cells were run for 10 cycles, and then they were disassembled, washed carefully with dimethyl carbonate and acetone, and dried. Figure 9a,d clearly show that the bare SnO_2_ electrode was easily broken, creating micro-holes on the surface. However, the M1SnO_2_ electrode was protected from washing, as illustrated in Figure 9b,e. This indicates that the MoS_2_ layer can facilitate the formation of a stable and smooth SEI layer. In Figure 9c,f, the M3SnO_2_ electrode after 10 cycles shows a thick MoS_2_ surface that is much rougher than that of the M1SnO_2_ surface. This would be due to the restacking of the MoS_2_ NS into a bulky multilayer state. Based on the results, a thin MoS_2_ coating layer acted as a protecting layer and formed a uniform SEI layer. The multiple MoS_2_ NS layers generated thick and rough surfaces, leading to inferior electrochemical performance for lithium-ion storage.

## 4. Conclusions

In summary, this study successfully prepared SnO_2_ NPs by a facile hydrothermal method and MoS_2_ NS using a liquid chemical exfoliation method. The materials were characterized by XRD, SEM, and TEM measurements. The size of the SnO_2_ NPs was about 5–10 nm, and the MoS_2_ NSs were hundreds of nanometers to a few micrometers in size. A new coating method utilizing the self-assembly of MoS_2_ NS thin films was successfully developed based on a rigid water–air interface. The addition of single-layer MoS_2_ NSs enhanced the cyclic stability of the SnO_2_ anodes for lithium-ion storage with a high coulombic efficiency of ~99% and high charge/discharge capacity of 281/277 mAh·g^−1^ compared to those with multiple MoS_2_ layers after 100 cycles. These results also suggest that single-layer MoS_2_ in large-scale fabrication methods, such as chemical vapor deposition, could be applied to further enhance the cyclic stability of anodes in LIBs.

## Figures and Tables

**Table 1:** Resistance values extracted from the equivalent circuit of M1/M2/M3SnO_2_ anodes.

Sample	R_s_ (Ω)	R_ct_ (Ω)	R_SEI_ (Ω)
SnO_2_ NPs	1.9	23.8	500.2
M1SnO_2_	2.4	59.3	182.7
M2SnO_2_	2.8	76.2	284.8
M3SnO_2_	2.8	86.1	305.1
